# Ethical dilemmas at the end of life: a reflection from the Philosophical Perspective of Luigina Mortari

**DOI:** 10.1590/0034-7167-2022-0759

**Published:** 2023-12-04

**Authors:** Vitória Pessoa Nogueira, Marília Alves Furtado, Vera Lúcia Mendes de Paula Pessoa, Viviane Magalhães Pereira

**Affiliations:** I Universidade Estadual do Ceará. Fortaleza, Ceará, Brazil

**Keywords:** Terminal Care, Bioethics, Nursing, Ethics, Philosophy, Cuidado Terminal, Bioética, Enfermería, Ética, Filosofía, Assistência Terminal, Bioética, Enfermagem, Ética, Filosofia

## Abstract

**Objectives:**

to reflect on the ethical dilemmas involved in the care of patients at the end of their lives.

**Methods:**

this is a theoretical-reflective study based on the ethics of care proposed by Luigina Mortari.

**Results:**

discussing care involves addressing the ways of being inherent to human existence and understanding the unique characteristics of this condition. Ethical care constitutes an action driven by interest in the other and by the perception of their need. Ethical dilemmas are a part of end-of-life care, making it essential to maintain respectful assistance that considers the patient’s autonomy, using strategies for expressing their wishes, and ensuring continuous clear and empathetic communication among all those involved in providing care.

**Final Considerations:**

issues related to being, stemming from one’s reality of dependency and vulnerability, contribute to the emergence of ethical dilemmas present in care actions.

## RESUMO

**Objetivos:**

refletir acerca dos dilemas éticos presentes no cuidado de pacientes ao fim da vida.

**Métodos:**

trata-se de um estudo teórico-reflexivo baseado na ética do cuidado de Luigina Mortari.

**Resultados:**

falar de cuidado é abordar os modos de ser pertencentes à existência humana e compreender as características próprias dessa condição. O cuidado ético constitui uma ação movida pelo interesse no outro e pela percepção de sua necessidade. Dilemas éticos fazem parte do cuidado ao fim da vida, sendo necessário manter uma assistência respeitosa que considere a autonomia do paciente, utilizando estratégias para a manifestação de sua vontade, e garantindo a continuidade de uma comunicação clara e empática entre os diversos sujeitos envolvidos no cuidado.

**Considerações Finais:**

as questões relacionadas ao ser, decorrentes de sua realidade de dependência e vulnerabilidade, contribuem para o surgimento dos dilemas éticos presentes nas ações de cuidado.

## RESUMEN

**Objetivos:**

reflexionar sobre los dilemas éticos presentes en la atención de pacientes al final de la vida.

**Métodos:**

este es un estudio teórico-reflexivo basado en la ética del cuidado de Luigina Mortari.

**Resultados:**

hablar de cuidado es abordar los modos de ser pertenecientes a la existencia humana y comprender las características propias de esa condición. El cuidado ético constituye una acción impulsada por el interés en el otro y por la percepción de su necesidad. Los dilemas éticos son parte de la atención al final de la vida, siendo necesario mantener una asistencia respetuosa que considere la autonomía del paciente, utilizando estrategias para la manifestación de su voluntad y garantizando la continuidad de una comunicación clara y empática entre los diversos sujetos involucrados en la atención.

**Consideraciones Finales:**

las cuestiones relacionadas con el ser, derivadas de su realidad de dependencia y vulnerabilidad, contribuyen al surgimiento de los dilemas éticos presentes en las acciones de cuidado.

## INTRODUCTION

From a phenomenological perspective, care is defined as a mode-of-being, connected to human existence as a caring existence. This idea is supported by Heidegger when he defines humans as “caring-beings”^(1)^. Luigina Mortari, an Italian epistemologist, employed phenomenology as a method to pinpoint the essence of care and to grasp the ways it manifests - the modes of being-there inherent to caring. She delves into the connection between care and the human condition and the ethical essence of this practice in the pursuit and realization of good.

On the essence of care, Mortari defines it as: “*a practice that unfolds in a relationship, taking place over a variable time span, fueled by an interest in the other and aimed at promoting their well-being. Thus, it addresses something essential for the other*”. In Mortari’s view, care is an act motivated by the intention to benefit another, brought to life through the stances and modes of being-there^(2)^.

Humans can never truly master or fully inhabit their existential state. Given this inherent fragility, the imperative for care remains unceasing. Thus, care pervades our existence comprehensively; from birth until our final moments, humans rely on the care of others, self-care, and care for their environment^(2)^.

For healthcare professionals, like nurses, the phenomenology of care has evolved into an ethical foundation for their work, positioning care as pivotal in the process of healing diseases and in supporting vulnerable patients^(1)^. When in good health, one can fully embrace life, exploring new facets of being-there. But when illness strikes, the volatility of existence, trapped between a past existence and an uncertain future, is felt intensely. The road ahead becomes unpredictable, imposed, and inevitable; at such junctures, reliance on others is often indispensable, frequently leading to individual suffering^(2)^.

Within this framework, care frequently exhibits an imbalance between the giver and receiver, especially during life phases marked by heightened vulnerability and reliance^(2)^. This imbalance often places healthcare professionals in dilemmatic positions when making decisions, particularly in end-of-life situations. Cultural and philosophical influences, coupled with societal individual and collective values, prompt ongoing debates and spawn ethical dilemmas in these professionals’ practice^(3)^.

These dilemmas are anchored in the core of patient care, touching upon the boundaries of therapeutic interventions. Issues might include the suitability of cardiopulmonary resuscitation for those in palliative care, the judicious use of medications like end-of-life antibiotics or continuous sedation, challenges with opioids - ranging from undertreatment to overuse and even opioid phobia - and navigating the complex waters of patient autonomy via advanced directives (AD), especially when they conflict with family wishes^(4)^.

Conflicting ethical situations in end-of-life patient care will invariably persist, as the healthcare landscape constantly evolves, demanding unwavering dedication from health professionals for nuanced understanding^(5)^. Philosophical reflection proves invaluable here, illuminating the intricate dance between care and humanity. The ethics of care stands out as a potent lens for interpreting and addressing ethical quandaries in end-of-life caregiving^(3,6)^. Thus, by recognizing care as an ontological entity, intrinsic to existence, and framing death as an innate part of the human journey, we aim to engage with the topic through Luigina Mortari’s ethics of care.

While this article occasionally zeroes in on nursing care, the insights and reflections presented have broader implications, encompassing other professional sectors involved in end-of-life caregiving, all of whom grapple with ethical dilemmas in this realm.

## OBJECTIVES

To reflect on the ethical dilemmas in the care of patients at the end of life, given that providing assistance during this period is challenging for both caregivers and those receiving care.

## METHODS

This theoretical-reflective study is based on the insights of Luigina Mortari, as presented in her work “Philosophy of Care” (2018)^(2)^. In this book, Mortari explores care from both an ethical and philosophical standpoint. She has written other works addressing the concept of care. In “Philosophy of Care”, Mortari employs phenomenology as a method and theoretical framework, drawing from the ideas of Martin Heidegger (1889-1976), Edith Stein (1891-1942), and Emmanuel Lévinas (1906-1995), as well as ancient Greek philosophers such as Aristotle (384-322 B.C.) and Plato (428/427-348/347 B.C.), among others. The article is structured around the following topics: Care and Being: The Ontology of Care; Ethical Care at the End of Life; and Ethical Dilemmas in End-of-Life Care.

## RESULTS

### Care and being: the ontology of care

The phenomenon of care is fundamental and indispensable for life; without it, life cannot flourish. The intertwined relationship between care and life underscores its inseparability, evident from birth when a human being cannot survive without the care of others. This dependency continues until life’s end, highlighting the continuous duty to care for oneself and others. Consequently, care can be perceived as the essence of living and existing. Thus, care is an ontological feature, recognized as the study of phenomena and an inherent existential condition, as it fosters the safeguarding and continuation of life. Yet, because care is so integral to existence, its ontological significance might often be overlooked, mirroring what transpires with what is ontologically nearest to man ^(2)^. This oversight arises because healthcare professionals frequently concentrate on action, techniques, and ways of caring, or the ontic manifestation of the care phenomenon.

Care invariably pertains to someone or something and carries with it an intent, manifesting as tasks, interest, concerns, and solicitude. This term typically describes nurses’ actions that focus on restoring or maintaining another’s well-being. Healthcare always implies attentiveness towards another, bearing a character of otherness, hence endorsing a collaborative inclination. In essence, to care is to act with reciprocity and compassion, assuming responsibility. Caring signifies becoming accountable, showing concern, and endeavoring for someone or something’s well-being. Care holds value for both its practitioners, who deem it vital for life, and those who benefit from it, as it enhances the richness of existence^(2)^.

Being serves as the foundational dimension that facilitates the manifestation of the entity, including Dasein itself (being-there). All human existence structures converge around care (1). Humanity isn’t self-contained; it’s perpetually marked by needs and necessities. Humans are neither completely autonomous nor wholly self-reliant. A person is always reliant on external factors, whether it’s the environment they’re born into, the context that shapes their existence^(2)^, culture, education, or public policies that may either facilitate or hinder the realization of their potential.

Given this innate ontological vulnerability, the mandate to care for another emerges. Hence, we discern three care dimensions, thereby revealing its polysemy. Care is directed towards conserving vitality and supporting existence; it also seeks to propel humans towards transcendence, nourishing them with purpose. Lastly, there’s restorative care, addressing both the physical and spiritual realms, activated when either body or spirit is afflicted. In every instance, care fulfills an ontological necessity^(2)^.

While delving into the ethics of care, it’s crucial to distinguish between natural care and ethical care. When we act voluntarily for another’s benefit, we exemplify natural care. A prime instance is a mother caring for her child, where no moral disparity exists between action and intent; therefore, an ethical dimension isn’t acknowledged. Conversely, when duty clashes with personal desires, demanding effort to materialize care, it denotes an ethical action. Such a distinction might imply that natural care lacks ethics. Nevertheless, the care dynamic is inherently asymmetrical, involving a caregiver and a recipient. Consequently, ethical dilemmas are integral to care^(2)^. Beyond discussions about human vulnerability, inconsistency, and dependency, it’s pivotal to underscore what drives a caring action: the focus on another’s needs. The caregiver, thus, commits to what’s indispensable to the recipient, something they can’t achieve independently^(2)^.

Luigina Mortari^(2)^, in juxtaposing ethics with ontology, proposes that the former emanates from a narrative transcending the individual, where collective well-being becomes paramount. Consequently, ruminating on care ethics emphasizes the concept of goodness. Ethical actions interwoven with care gravitate towards life-affirming benefits, making life enriching and enjoyable. From a caregiver’s standpoint, advocating for the good entails promoting the recipient’s well-being. For Mortari, the ultimate goal of care is life quality. Exceptional care is proactive, seeking goodness, and protective, striving against all harm.

From the insights shared, the inherent relational essence of care becomes transparent, culminating in existential implications for every participant in the process. Additionally, ethical care, which inherently pursues goodness, is a constant in human interactions. Given man’s unique caring existence, the subsequent section will explore facets vital for ethical care at life’s twilight.

### Ethical care at the end of life

The ethics surrounding care at the end of life become particularly pronounced due to the evident dependence and significant asymmetry between the caregiver and the recipient. Often, the individual receiving care might experience reduced autonomy or even a complete loss, as seen in those under deep sedation. In such circumstances, one fundamental pillar of ethical care is respect. It’s imperative to pay close attention to the patient’s expressed values and wishes, devoid of any preconceived notions, and to uphold the dignity of the person in the care-receiving position^(2)^.

Engaging with the patient through care, expressing tenderness, and being receptive to their subjectivity embodies respect. Before undertaking any action or medical procedure, it’s essential to ensure the patient has a voice and is genuinely heard, recognized in their unique manner of existing in the world^(2)^.

Furthermore, it’s vital to acknowledge that death isn’t merely an end for humans but a defining and forthcoming facet of existence. The adverse perception of death stems from treating the act of dying as a mere footnote in human existence. By embracing mortality, recognizing it as intrinsic to life rather than its mere conclusion, one finds that dying holds profound meaning. At every stage, humans exist and live within limits^(1)^. Within this framework, end-of-life care assumes an ethical duty: facilitating the patient’s introspection on their existential boundaries, understanding their finite nature, enabling their spiritual transcendence, and attending to both physical and spiritual wounds.

With this perspective, caregivers must be attuned to the unique needs of the individual under their care. Beyond just tending to physical necessities, it’s crucial to ensure that the individual’s subjectivity isn’t overshadowed, especially during times of heightened dependence. It’s a fine balance to strike between genuine care and overbearing paternalism, and between what is vital versus what might be redundant for the patient’s well-being^(2)^. The complexities intensify in terminal care situations, where diverse and highly individualized palliative needs span biological, social, spiritual, and religious realms^(4)^.

Detecting the core needs for someone’s well-being at life’s end can be intricate. The optimal approach is to encourage patients to articulate what they believe constitutes a dignified and peaceful existence. For those unable to express themselves, tools like Advance Directives (ADs) can be invaluable. These serve as avenues to capture and honor the patient’s unique wishes concerning healthcare decisions. Crafting such directives demands a clear understanding of the illness and open dialogue with health professionals to eliminate uncertainties regarding potential treatments and interventions, thereby aiding the patient in making enlightened choices^(7)^.

Intrinsically, ADs possess a relational essence. They necessitate medical professionals’ guidance to elucidate potential clinical scenarios, aiding patients in making well-informed decisions. Mortari^(2)^ observes that every individual wields an autonomy that’s never absolute, given our interdependence on others. Care, in Mortari’s view, emerges as an ontological event embedded in interrelations. When one commits to another’s essential needs, care transpires. Thus, capturing a patient’s desires through ADs, and respecting those wishes, can be seen as acts of care, addressing pivotal concerns of the patient.

However, ADs come with their set of limitations. For instance, in the Brazilian context, the resolution defining ADs and guiding their creation remains somewhat ambiguous. It fails to precisely delineate what can or can’t be included concerning a patient’s preferences. Foreseeing and detailing measures based on evolving clinical conditions, especially those manifested towards life’s end, is challenging. Consequently, the written documentation seems more angled towards affirming ADs’ legal stature rather than capturing the nuanced essence of a patient’s wishes^(2)^.

In this way, Advance Directives (ADs) possess a relational nature, as they require professional intervention for clarifications about the clinical conditions that can be included in the document. This assists the patient in making an informed decision. Mortari^(2)^ emphasizes that each individual carries an autonomy that is never absolute since it depends on others. The author views care as an ontological phenomenon, intrinsic to the relationships between beings, which occurs when someone dedicates themselves to something vital for another. Thus, recording the patient’s preferences and wishes through ADs, and duly considering them, constitutes forms of care, as they address essential aspects for the patient.

However, several limitations are associated with ADs, such as their broad nature in relation to the points presented in the Brazilian resolution that defines and provides guidelines for their creation. For instance, it is not clearly specified which elements can or cannot be included concerning the patient’s wishes. It can also be challenging to detail in advance the measures that should be taken based on the patient’s clinical condition, which is often determined near life’s end. Thus, the written document appears more focused on ensuring the legality of the ADs than on capturing the patient’s true intentions^(7)^.

The challenge with ADs, related to anticipation, stems not only from the unpredictability of circumstances but also from the individual drafting them, that is, the patient. This can introduce distress and concerns. For Mortari^(2)^, the ability to project, an inherent characteristic of existence, should be approached with an unpredictable future in mind. Furthermore, during an illness, envisioning the future can become painful, as the sense of projection diminishes in the face of suffering. In this scenario, nurses may grapple with dilemmas over which actions best serve the patient. This line of thinking might suggest that if the creation of ADs induces suffering, it might be viewed as unethical care, even if the intention was to benefit the patient.

Given the potential to actualize or establish end-of-life care choices through ADs, it’s advisable not to draft them during periods of intense suffering. Such times might render them less than ideal for the patient, potentially evoking negative emotions. Thus, not everyone would benefit from proactive considerations about their mortality. However, it’s vital to recognize that these actions might facilitate a broader understanding of the being-towards-death concept, thereby reducing potential ethical dilemmas in nursing care.

When drawing distinctions between necessary and futile actions, one delves into the realm of ethical care, a domain that seeks to avoid dysthanasia. A cornerstone of palliative care is the principle that neither hastens nor postpones death; instead, it permits death to unfold naturally^(4)^. Dysthanasia denotes the undue prolongation of a patient’s life, which subjects them to unnecessary suffering. Given the rapid advances in science and technology, this has emerged as a pressing ethical dilemma in contemporary times^(8)^. According to Mortari^(2)^, the essence of ethical care inherently gravitates towards the greater good, characterized by its proactive and safeguarding nature. Consequently, in the context of ethical care, aggressive therapeutic interventions that merely extend life-yet amplify pain and suffering-are shunned.

When we conceive of care as a relational endeavor aimed at promoting another’s well-being^(2)^, ethical care, in these contexts, shouldn’t focus on extending human suffering. Instead, it should prioritize enhancing the quality of life for the time remaining. Such care values the individual’s perspectives and encompasses actions like pain relief, comfort maintenance, closeness to loved ones, upholding the person’s dignity, and fostering a sense of peace. This peace can be attained through alleviating anxieties, stressors, and fears^(9)^, or by facilitating spiritual encounters and redefining relational roles. Therefore, ethical care, which inherently seeks the greater good, is rooted in intentional gestures and actions. The challenge is pinpointing the right actions, at the optimal moment, tailored to a specific individual - actions that genuinely benefit the recipient^(2)^.

This calls for ongoing reflection and thoughtful deliberation, ensuring that professionals continuously scrutinize their actions. It’s crucial that the technical aspects don’t eclipse the unique needs of each person. To truly pursue the greater good, one must not only think rationally but also genuinely feel the presence of the other and show genuine concern, especially during moments of heightened existential vulnerability, such as life’s end. After delving into the ethics of care during this phase, the ensuing discussion will address the challenges inherent in end-of-life care that give rise to ethical quandaries.

### Ethical dilemmas in end-of-life care

An ethical dilemma arises when one must choose between alternatives that are equally desirable or undesirable. This necessitates analysis, discussion, and reflection for each situation before a decision is made^(2)^. Such dilemmas are often perceived as an inevitable aspect of healthcare, often going beyond deontological contexts, thereby encompassing the personal experiences of each professional^(6)^.

Beyond the patient, dilemmas might also pertain to other team members, the family, dynamics within the healthcare institution, and even national policies. Autonomy is a primary concern associated with ethical dilemmas. Healthcare professionals, for instance, must decide whether it’s appropriate to share or withhold information about a diagnosis or poor prognosis. They face conflicts with patient autonomy when such information remains undisclosed^(10)^. Thus, being truthful about a severe diagnosis and/or prognosis poses a challenge for healthcare professionals. Possessing the ability to communicate effectively is paramount for those providing care to end-of-life patients.

According to De Panfílis, Di Leo, Peruselli, and colleagues, clear and effective communication is pivotal in interpersonal relationships within the healthcare environment. It aids professionals in recognizing and tackling moral dilemmas that may emerge during decision-making. Provided it respects the patient’s wishes and preferences, candid communication can enhance symptom control and quality of life^(6)^.

Communication that not only addresses the patient’s clinical status but also facilitates the exchange of values and emotions fosters bonds of respect, easing the decision-making process. Often, the responsibility of making healthcare decisions can introduce stress and anguish for both families and patients. This underscores the importance of dialogue and the capacity to navigate the vast psychosocial and spiritual needs of the patient and their families^(4)^. As Mortari^(2)^ points out, the deeper the vulnerability and dependence of an individual, the more intense is the sense of responsibility felt by those deciding on their behalf. Caregivers also grapple with the repercussions of their choices, recognizing that their success can be influenced by external factors.

Expanding on situations that trigger dilemmas in end-of-life care, families may advocate for clinical interventions that professionals deem not optimal for the patient. Determining the right moment to transition from disease-altering treatments to symptom management, coupled with divergent views among multidisciplinary team members, palliative care units, and other specialties, compounds these dilemmas^(10)^.

In the context of decisions that professionals deem unsuitable, Mortari’s^(2)^ insights into caregiving situations offer valuable perspectives. She posits that when an individual mistakenly perceives a need as essential, it’s incumbent upon caregivers to help them understand that not acceding to their request is, in that particular moment, an act of care. However, navigating this stance requires caution to ensure decisions don’t become coercive or oppressive^(2)^. This viewpoint further suggests that caregiving mandates a forward-thinking approach, especially when confronted with therapeutic options that could be harmful to the patient.

Confronted with such dilemmas, nurses often rely on their values, empathy, and dialogues with colleagues. They factor in the family’s perspective and honor the patient’s end-of-life desires. In this journey, understanding ethical principles is just the beginning. Collaborative efforts with the multi-professional team are vital, aiming not only to address dilemmas but to humanize patient care^(5)^.

The act of caregiving can demand courage, particularly when challenging established therapeutic decisions. Mortari^(2)^ illustrates this with the story of a nurse confronting a doctor about a terminal melanoma patient in relentless pain due to the prescribed therapy. This dispute prompted the doctor to reevaluate the nurse’s concerns in consultation with other professionals. As a result, a modified pain therapy was introduced, bringing considerable relief to the patient, who could then rest for extended periods.

Ethics is not only required when facing major choices. Dilemmas arise in every moment of interaction with others, and decisions of great importance to one’s life must be made. It’s essential to remain attentive and sensitive to others to ensure sound moral deliberation. According to Mortari^(2)^, individuals who perform significant caregiving gestures, like the nurse mentioned by the author, respond to what they perceive as essential to the other person. They recognize human fragility and understand the non-negotiable aspects. Therefore, when making an ethical decision in the face of a dilemma, one must observe the presented reality and attentively seek the meaning of “being there,” guided by a passion for the other’s well-being^(2)^.

These aspects are fundamental to the discussion of ethical care. Even when confronted with matters of profound ethical value, moral reasoning is not merely a cold, neutral faculty; it is sensitive^(2)^. Elements mentioned in the ethics of care are not found in approaches grounded in classical bioethical principles, such as autonomy, beneficence, non-maleficence, and justice. Literature raises criticisms about a perspective that views humans as beings full of consciousness, competence, and independence but fails to address the complexities of interpersonal relationships and the presence of emotions in moral perceptions and decision-making processes^(6)^.

## FINAL CONSIDERATIONS

From the insights presented, it becomes evident that concerns surrounding the nature of human existence, stemming from one’s inherent dependence and vulnerability, give rise to ethical dilemmas in caregiving actions. As such, the ethics of care emerge from relationships between individuals and from a transindividual perspective. Here, ethical care becomes the product of actions tailored to the needs of another, centering on their well-being and enhancing their overall quality of life.

The intrinsic asymmetry in caregiving, particularly pronounced in end-of-life situations, calls for a deeper understanding of another person’s way of life. This, in turn, creates opportunities for exploring new modes of existence, even in the face of impending death. Valuing the uniqueness of each individual becomes paramount, with advanced directives serving as a key facilitator for ethical care. It is crucial to recognize and account for their potential needs, which can lead to emotional distress for the patient and possible conflicts in caregiving scenarios.

Ethical conundrums frequently arise in end-of-life care contexts. When nurses discern the reality and needs of others, their actions are driven by the well-being of the patient. At times, personal values also inform their decision-making processes. This discourse accentuates the pivotal role of care ethics and the insights of Luigina Mortari in illuminating the complexities of caregiving and the broader nuances of human existence. In light of this, caregivers attending to patients at the end of their lives must consistently engage in deeper reflection and understanding. This commitment not only enhances the quality of a patient’s life but also mitigates potential ethical tensions in their caregiving practices. Ultimately, end-of-life ethical care should aspire not to prolong human suffering but to elevate the quality of life for as long as it persists.
